# Treatment of facial squamous cell carcinoma in a centenarian using a combination of surgery and photodynamic therapy: A case report

**DOI:** 10.1097/MD.0000000000045431

**Published:** 2025-10-24

**Authors:** Xuefeng Fu, Kaili Chen, Meiyan Wang, Min Zhang, Qi Tu, Lujian Zhu, Qiying Zhang

**Affiliations:** a Department of Dermatology, Affiliated Jinhua Hospital, Zhejiang University School of Medicine, Jinhua, China; b Department of Neurosurgery, Affiliated Jinhua Hospital, Zhejiang University School of Medicine, Jinhua, China; c Department of Infectious Diseases, Affiliated Jinhua Hospital, Zhejiang University School of Medicine, Jinhua, China.

**Keywords:** 5-aminolevulinic acid-based photodynamic therapy, centenarian, cutaneous squamous cell carcinoma, malodorous wounds, open-wound treatment

## Abstract

**Rationale:**

Cutaneous squamous cell carcinoma (cSCC) is the second most common nonmelanoma skin cancer. Treating aggressive cSCC in very elderly patients, especially centenarians with significant health issues, poses a major challenge due to the risks of standard surgical excision under general anesthesia. This report presents a novel palliative approach that combines limited surgical resection, open-wound management, and photodynamic therapy (PDT) for facial cSCC in a centenarian, suggesting it as a safe and effective alternative when conventional treatments are unsuitable.

**Patient concerns:**

A 101-year-old female patient presented with a rapidly enlarging cutaneous mass on the left side of her face, characterized by erosive lesions and a malodorous scent. The patient reported no history of trauma or significant sun exposure.

**Diagnoses:**

A computed tomography scan of the orbit identified a soft tissue shadow indicative of a mass located in the lateral aspect of the left orbit, whereas the remaining structures of both orbits appeared unremarkable, with intact bony walls. Clinical manifestations were suggestive of squamous cell carcinoma. Histopathological examination of the postoperative lesion tissue, utilizing hematoxylin-eosin staining, revealed a significant presence of squamous cells, along with scaly vortices, keratin pearls, and nuclear atypia, thereby confirming the diagnosis of squamous cell carcinoma.

**Interventions:**

The patient underwent a surgical resection in combination with PDT. Specifically, a straightforward surgical resection was executed with a 5-millimeter margin. The surgical incision was intentionally left open and not sutured. During the management of the open wound, PDT was administered.

**Outcomes:**

The patient elected to undergo a surgical resection and a single PDT session, after which the wound gradually healed from the edges, drying without infection. Over 2 months, the wound healed by secondary intention, leaving a flat, stable scar. The patient was very satisfied with the cosmetic result and odor resolution. At a follow-up 7 months later, and until the patient’s death from unrelated causes, there was no sign of local recurrence.

**Lessons:**

We suggest that surgery combined with open-wound and PDT could be a safe and effective treatment for squamous cell carcinoma in elderly patients and those with comorbidities who cannot undergo general anesthesia.

## 1. Introduction

Cutaneous squamous cell carcinoma (cSCC) is the second most common nonmelanoma skin cancer. It arises from the malignant proliferation of keratinocytes originating in the epidermis and associated adnexal structures, such as pilosebaceous units and eccrine glands, and typically manifests clinically as an indurated, crusted lesion.^[1]^ Certain invasive cSCCs can cause severe cosmetic damage with malodorous wounds. For the most invasive, high-risk cSCCs, surgical excision is necessary, and this can include either conventional wide local excision or Mohs micrographic surgery, depending on the tumor features and the anatomical location^[2]^

As aging is a physiological phenomenon in which the functional capacity of organs and tissues decreases due to degenerative changes in the structure, and aging is always accompanied with comorbidity, management of centenarian patients with cSCC requires special considerations and understanding and faces enormous challenges.^[3]^

In the treatment of skin tumors, photodynamic therapy (PDT) involves activating photosensitizers, most commonly 5-aminolevulinic acid (ALA) or methyl aminolevulinate (MAL) using visible light. When activated, these agents generate reactive oxygen species within target cells, triggering tumor cell apoptosis or necrosis. Additionally, PDT disrupts the tumor’s microvasculature, leading to hypoxia, contraction of tumor-associated blood vessels, and thrombosis. These effects culminate in the complete occlusion of tumor-nourishing microvessels, ultimately causing ischemic necrosis of the tumor tissue. PDT owing to its accessibility and the added benefit of favorable cosmetic outcomes, has been widely adopted for both the treatment and prevention of cSCC.^[4]^ Clinical studies have demonstrated that after 1 to 2 cycles of MAL-PDT, lesion clearance rates for cSCC reached 88% to 100% at 3 months posttreatment. During follow-up periods of 17 to 50 months, 68% to 89% of treated lesions remained recurrence-free.^[5]^ However, the European dermatology forum guidelines for topical PDT highlight the currently limited evidence on the efficacy of PDT for invasive cSCC, explicitly stating that PDT monotherapy is not recommended for treating such invasive squamous cell carcinoma cases. Here, we present a case report of a 101-year-old female patient with aggressive cSCC on the face. Following treatment with surgical excision combined with PDT, the wound progressively healed spontaneously, accompanied by complete resolution of the foul odor.

## 2. Case presentation

A 101-year-old woman presented with a skin mass on the left side of her face that had shown gradual enlargement with erosive and malodorous trauma. The lesion had appeared 8 months prior to the visit and was growing rapidly. The centenarian patient had been previously diagnosed with skin tumor and skin infection in the local hospital, which instructed her to use Compound Polymyxin B Ointment externally. However, the mass did not improve, and a malodorous smell gradually appeared.

**Figure 1.**
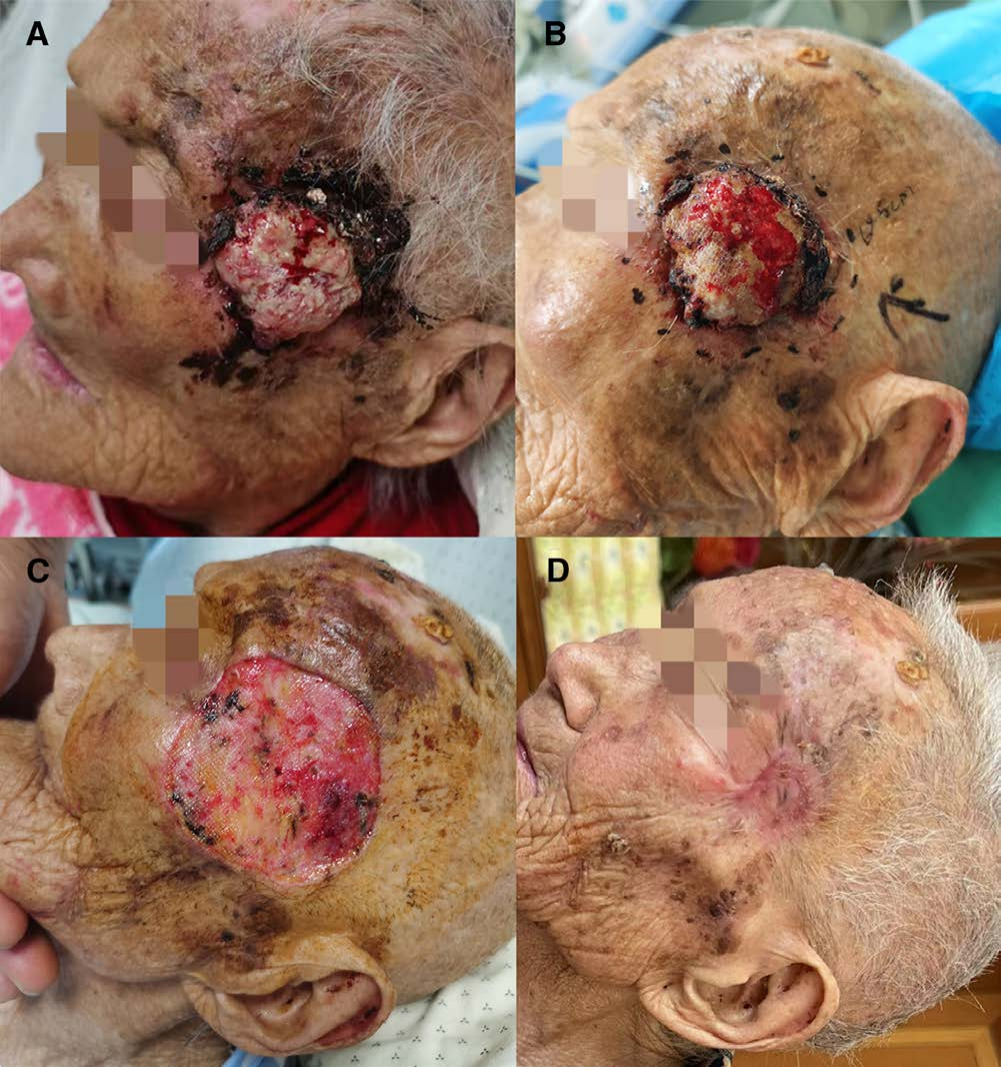
Treatment process. (A and B) The skin lesion after admission; (C) exposed wound; (D) 6 mo after tumor resection.

**Figure 2.**
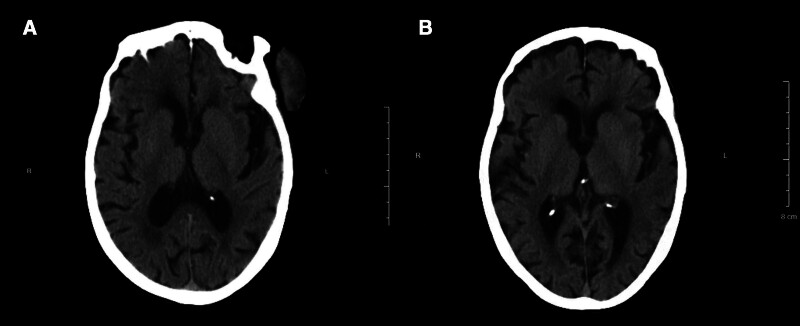
CT scan of the orbit. (A) CT scan after admission; (B) CT scan 6 mo after tumor resection. CT = computed tomography.

The patient had no history of trauma or excessive sun exposure. Physical examination revealed a 4.5*4 cm-sized skin mass on the left lateral orbital side of the face, raised above the skin with surface erosion accompanied by pus (Fig. 1A and B). A computed tomography scan of the orbit revealed a mass-like soft tissue shadow in the left lateral orbital region, while the remaining structures of both orbits were clear with continuous bone walls (Fig. 2A). The clinical manifestations suggested squamous carcinoma. The patient had a history of atrial fibrillation, cardiac insufficiency, and pleural effusion. Considering the presence of these underlying diseases, the patient’s limited life expectancy, and the high risk of anesthesia, we advised the patient to forego surgical treatment and receive palliative care instead but the patient refused. While it would have been possible to use palliative radiation radiotherapy or photodynamic therapy instead of surgery, the patient refused these options too. The patient’s residence was situated at a considerable distance from the hospital. Undergoing palliative radiotherapy or photodynamic therapy alone necessitated multiple journeys between home and the hospital. The patient’s weakened physical condition rendered her unable to endure such frequent travel, and the associated costs were relatively high, imposing a significant financial burden. Furthermore, radiotherapy or photodynamic therapy alone yielded limited improvement in malodorous smell and aesthetic appearance, with outcomes falling short of those achieved through prior surgical excision. The patient was keen to undergo surgical resection and was even willing to accept the risk of death during surgery. Her insistence on resection was based on her wish to improve the aesthetics and alleviate the malodorous smell.

The risk of surgery under general anesthesia was too high for a centenarian with cardiac insufficiency, atrial fibrillation, and pleural effusion. According to the ASA Physical Status Classification System, an anesthesiologist classified the patient as ASA IV, representing a patient with severe systemic disease that is a constant threat to life. The size of the mass was 4.5*4 cm, too large for simple excision and suture. We told the patient and her family members that the safety margin for a cSCC of this size was at least 9 mm according to the clinical practice guidelines of the American Academy of Dermatology; however, the patient and her family members expressed concern about the difficulty of healing and infection resulting from a too-large incision and asked for palliative surgical treatment to preserve as much healthy skin as possible. The patient was informed that the malignancy might recur if the incision was not expanded to a safe extent. She accepted this risk and asked for palliative surgical treatment, stating that she essentially wished to remove the lesion to improve the aesthetics and minimize the smell.

The patient, who remained mentally alert and competent, was keen to undergo surgical resection. After multidisciplinary evaluation and extensive communication with the patient, we determined a treatment plan involving a simple surgical excision with a 5-millimeter margin, followed by a combination of open-wound and PDT. Reasons for selecting PDT as an alternative treatment modality include its comparatively lower invasiveness, high tissue selectivity, absence of serious adverse effects besides pain, good cosmetic outcome, low recurrence rate, and, more importantly, safety for elderly patients and patients with comorbid conditions. Ultimately, the patient consented to the treatment plan. The cardiology department recommended cardiac-strengthening (Digoxin) and diuretic therapy (Aldosterone) to achieve a state where the patient experienced no significant chest tightness while in a semi-recumbent position.

The patient was monitored electrocardiographically throughout the procedure, which was done under the supervision of an anesthesiologist. During surgery, after local infiltration anesthesia with lidocaine, the skin and subcutaneous fat layer were incised in a 0.5 cm ring along the margin of the mass, with separation along the subcutaneous fat layer and fascial surface. It took only 20 minutes to complete the surgical removal. The surgical wound was left open without repair (Fig. 1C). The original plan for the patient was to undergo 4 sessions of photodynamic therapy, repeated weekly, followed by a second-stage implantation.

During the open-wound period, the patient underwent 5-aminolevulinic ALA-PDT on the second postoperative day to promote wound repair and further lower recurrence rate. The wound was approximately 5*5.5 cm in size. ALA hydrochloride powder (6*118 mg) was dissolved in normal saline at a concentration of 20%. A gauze pad soaked in the ALA solution was applied topically to the lesion, after which the gauze pad was covered with a dark plastic sheet for 3 hours to prevent light from reaching the lesion. The wound was then exposed to irradiation with 635-nm LED light (power intensity 96 J/cm^2^, 80 mW/cm^2^) for 20 minutes. The selection of these treatment parameters was based on prior human studies demonstrating efficacy and safety of PDT for cSCC.^[6]^

**Figure 3.**
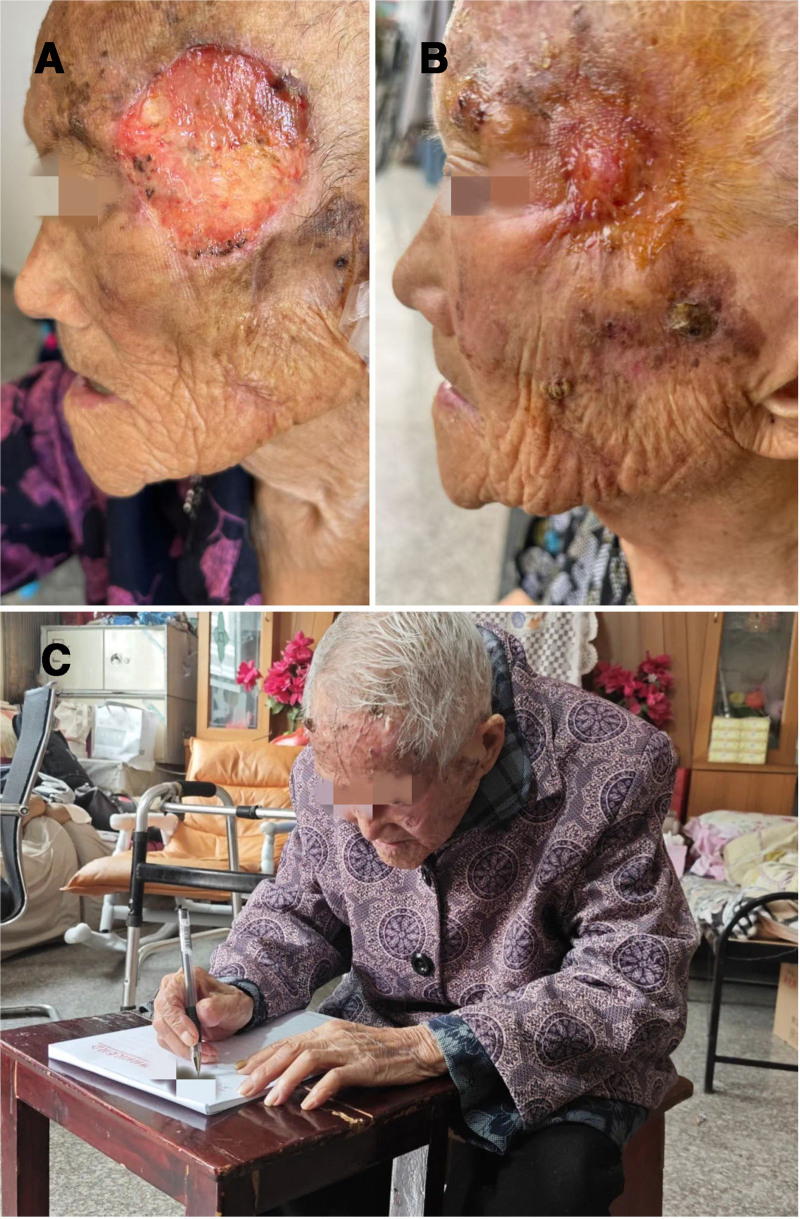
Pathological results showing a mass of squamous cells with scaly vortices, horn pearls, and nuclear atypia. (A) HE ×40; (B) HE ×100. HE = hematoxylin–eosin.

Histological analysis of the excised lesion using hematoxylin-eosin staining showed the presence of a mass of squamous cells with scaly vortices, horn pearls, and nuclear atypia (Fig. 3A and B), confirming the diagnosis of squamous cell carcinoma. No residual tumor cells were found around the cutting edge.

**Figure 4.**
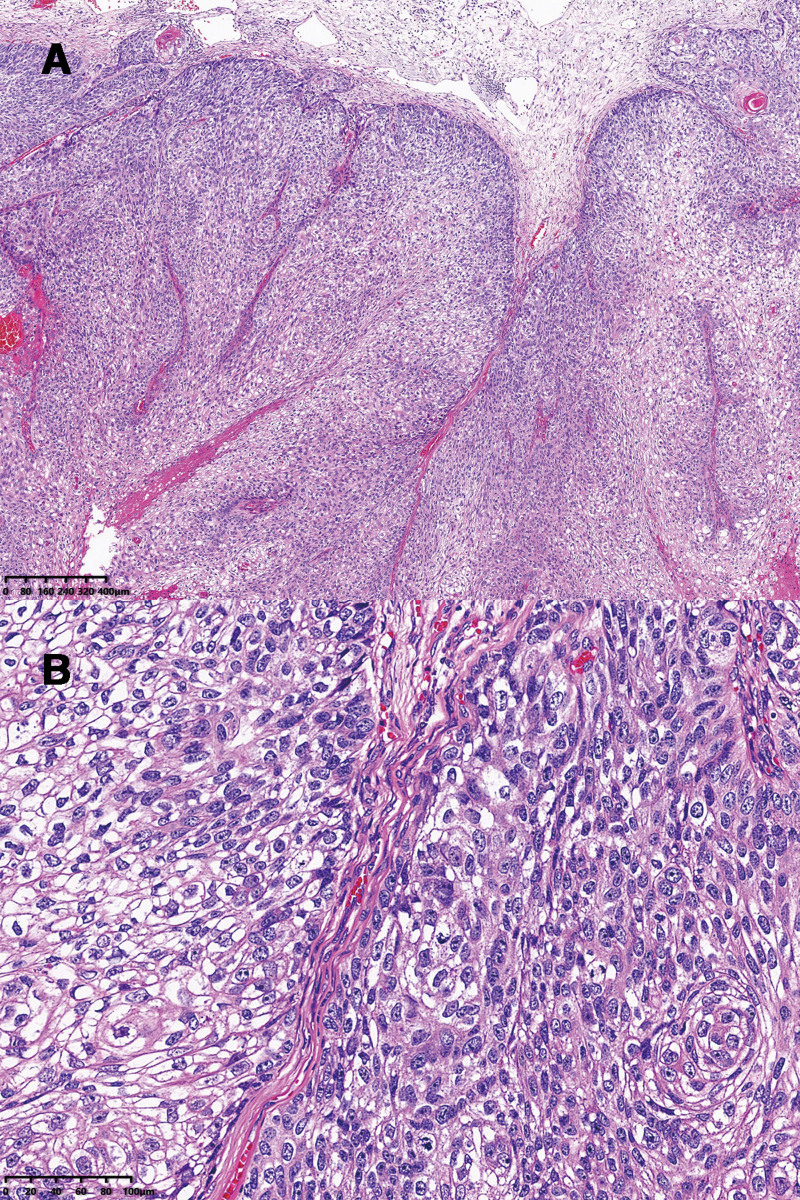
Further details of the wound-healing process and a picture of the patient. (A) Exposed wound 2 wk after tumor resection; (B) exposed wound 2 mo after tumor resection; (C) a picture taken when she practiced handwriting 6 mo after tumor resection.

The patient chose to undergo only 1 session of photodynamic therapy due to personal reasons. Surprisingly, during the process of recuperation and waiting, the wound became dry and healed gradually on its own (Fig. 4A and B), and the malodorous smell disappeared completely. A second-stage implantation was not needed. A computed tomography scan of the orbit revealed no tissue shadow in the left lateral orbital region at the 6-month follow-up after the photodynamic therapy (Fig. 2B), with only a flat scar being visible (Fig. 1D). Her family reported that she could comb her hair, wash her face, eat independently, and had hobbies such as practicing handwriting (Fig. 4C). During a telephone follow-up appointment 7 months after treatment, we were informed by family members that the patient had just passed away. The local doctor subsequently reported that she had died of a lung infection and multiple organ failure during the winter, and that the skin on her left lateral orbital side was still intact at the time of her death, with no malodorous smell and no visible recurrence of squamous cell carcinoma of the skin.

## 3. Discussion

cSCC is the second most common form of nonmelanoma skin cancer (NMSC). The reported incidence of this cancer has increased recently, mainly due to population aging and the focus on skin cancer screening.^[7]^ However, there are few reported cases of this cancer in centenarians. To our knowledge, only 2 cases of cSCC in centenarian patients have been reported in the English literature, with the present report being the third to date.^[8]^ Some invasive cSCCs can cause severe cosmetic damage with malodorous wounds. Malodorous wounds can be distressing for patients and their families, negatively impacting quality-of-life outcomes. For health professionals, these wounds can also cause distress manifesting in feelings of disgust when faced with a wound emitting an unpleasant or repulsive odor.^[9]^

Here, we present a case of a 101-year-old female patient with an invasive cSCC lesion on her face accompanied by erosion and malodor. A careful pre-anesthetic evaluation was performed before surgery. The patient had cardiac insufficiency, atrial fibrillation, and pleural effusion which precluded excision of the tumor under general anesthesia with repair of the defect using reconstruction with flaps or skin transplantation. Considering the presence of these underlying diseases, the patient’s limited life expectancy, and the high risk of anesthesia, we advised the patient to forego treatment and undergo palliative care but the patient refused. Although radiotherapy or simple photodynamic therapy could be used as a palliative treatment,^[10]^ these options were refused by the patient.

The patient was willing to undergo surgical excision, and we used open-wound treatment after excision combined with PDT to promote wound healing. We consider that open-wound treatment is worth using for cSCC lesions at high-risk sites, and the treatment has been used previously.^[11]^ The 5-mm resection border of the mass did not meet the requirements of guidelines for this type of invasive squamous carcinoma, resulting in a certain risk of recurrence. The wound was left open after resection without implantation surgery, leading to risks of difficulties in wound healing, infection of the traumatized skin, and even the risk of further bloodstream infection. Our deliberations addressed ways to completely remove the mass, promote wound healing, prevent infection, minimize the probability of recurrence, and satisfy the patient’s desire for aesthetic improvement and elimination of the malodorous smell under the best possible circumstances and with the best possible protection of the patient’s life. It was important to shorten the surgical resection time as much as possible. Thus, the resection procedure took only 20 minutes to complete and the surgical wound was left open without repair.

Considering that the photodynamic guidelines and related literature mention that photodynamic therapy for squamous skin cancer has certain therapeutic effects on wound-healing, improving wound aesthetics, and postoperative scarring, we suggested the use of photodynamic therapy after surgery. The original plan was that the patient would undergo 4 sessions of photodynamic therapy, repeated weekly, followed by a second-stage implantation. The patient was discharged and returned home to recuperate after 1 photodynamic therapy session after surgery. The patient did not inform us of the reason why she chose not to continue with the second session of photodynamic therapy. We suspected that it might have been related to factors such as the long distance from the patient’s home to the outpatient clinic for retreatment, especially considering her age, as well as the fact that the relatively expensive cost of photodynamic therapy for treating squamous skin cancer was not yet covered by medical insurance in China, and also the consideration that photodynamic therapy was associated with a certain amount of pain, which the patient might not have wanted to undergo again. According to our previous experience, a wound area of 5*5.5 cm after open surgery does not heal easily by itself within 2 months with only simple sterilizing and dressing of the open wound, and even the second-stage implantation surgery is associated with a risk that the skin flap will not survive. Surprisingly, the patient’s wound healed gradually on its own during home recuperation after 1 surgical excision and 1 session of photodynamic therapy. No recurrence was observed during the 7-month follow-up after the treatment and only a flat scar (which was significantly reduced in comparison with the open-wound area) was observed on the face. The patient’s quality-of-life was thus greatly improved.

PDT is increasingly used due to its safety, efficacy, and excellent cosmetic outcomes, and is effective in promoting wound healing and preventing tumor recurrence.^[6]^ PDT has been instrumental in facilitating wound healing, perhaps through avoiding the development of infections by inactivating microorganisms such as bacteria and through promoting cell proliferation through the activation of stem cells which regulates inflammatory factors and collagen remodeling.^[12]^ To our knowledge, the present report is the first reported case where surgery followed by open-wound treatment with photodynamic therapy has been used for treating facial squamous cell carcinoma in a centenarian in the literature. With reference to authoritative journals, the surgical experience of our team, and the patient’s postoperative recovery, we concluded that the reason why the wound healed on its own without the need for second-stage implantation and no recurrence during 7 months of follow-up might be related to a certain extent to the postoperative photodynamic therapy. Compared with those of other palliative or minimally invasive treatments for cSCC in elderly patients, our treatment halted the progression of cSCC, improved the aesthetics and minimized the malodorous smell.

Therefore, we believe that for elderly patients with facial squamous carcinoma occurring in the presence of underlying diseases, open-wound treatment can be considered if the patient strongly desires surgery. We hope that this case can provide some new information for the future application of photodynamic therapy in older patients needing surgical resection, enabling the successful combination of cSCC resection with a good prognosis while respecting the patient’s wishes. This study is subject to several limitations that warrant consideration. Firstly, as a single-case report, the generalizability of the findings is inherently constrained. The favorable outcome observed may not be replicable across all similar patient populations. Secondly, the patient underwent only 1 session of the initially planned 4 sessions of ALA-PDT due to personal reasons. Although the outcome was successful, the full impact of the complete PDT regimen on wound healing and recurrence prevention remains indeterminate. Thirdly, the follow-up period was restricted to 7 months, concluding with the patient’s death from unrelated causes. A longer follow-up would have been necessary to more definitively evaluate long-term recurrence rates. Lastly, the treatment strategy was significantly influenced by the patient’s strong personal preferences, which may not align with a standardizable protocol. Future research involving a larger cohort of patients and extended follow-up periods is essential to validate this combined therapeutic approach.

Surgery followed by the combination of open-wound and photodynamic therapy may thus be a safe and effective method for the treatment of squamous cell carcinoma in elderly patients and patients with comorbid conditions for whom surgery under general anesthesia is inappropriate.

## Author contributions

**Conceptualization:** Xuefeng Fu.

**Data curation:** Kaili Chen, Qi Tu.

**Formal analysis:** Meiyan Wang, Min Zhang.

**Funding acquisition:** Xuefeng Fu.

**Investigation:** Lujian Zhu, Qiying Zhang.

**Resources:** Xuefeng Fu, Kaili Chen.

**Writing – original draft:** Xuefeng Fu, Kaili Chen.

**Writing – review & editing:** Qiying Zhang.
